# The Danish neonatal clinical database is valuable for epidemiologic research in respiratory disease in preterm infants

**DOI:** 10.1186/1471-2431-14-47

**Published:** 2014-02-17

**Authors:** Sofia Andersson, Jesper Padkær Petersen, Tine Brink Henriksen, Finn Ebbesen

**Affiliations:** 1 Department of Paediatrics, Aalborg University Hospital, Reberbansgade, DK 9000 Aalborg, Denmark; 2 Department of Paediatrics, Aarhus University Hospital, Skejby, Brendstrupgårdsvej 100, DK 8200 Aarhus N, Denmark

**Keywords:** Respiratory variables, Very preterm infants, Validation, Epidemiology, Quality assurance

## Abstract

**Background:**

We examined the quality of the information on the use of surfactant and the use of and duration of nasal continuous positive airway pressure (nCPAP), oxygen supplementation, and mechanical ventilation in the Danish Neonatal Clinical Database (NeoBase).

**Methods:**

We included all neonates born with a gestational age < 32 weeks admitted to a Neonatal Intensive Care Unit (NICU) at two university hospitals in 2005. On discharge, the clinicians complete a structured form with information related to the delivery and course of stay in the NICU. These forms were entered into the NeoBase. The nurses’ daily bedside documentation was used as reference standard. Concordance was used as a measure of agreement between the NeoBase and the reference standard. For the dichotomous variables the concordance was defined as the sensitivity of the information registered in the NeoBase. For the continuous variables, it was based on the discrepancy in days between the NeoBase and the reference standard. The percentage of concordance was described as high (> 90), moderate (70–90) or low (< 70).

**Results:**

Overall, 153 infants participated in the study. Concordance was high for all dichotomous variables. The NeoBase slightly underestimated the duration of nCPAP and mechanical ventilation. The duration of oxygen therapy was neither over- nor underestimated in the NeoBase. Concordance was low for all continuous variables if we assumed that the registered information was identical. It was 100% for duration of mechanical ventilation and moderate for nCPAP and oxygen supplementation if we allowed for a discrepancy of 1 day.

**Conclusion:**

The NeoBase is a valuable tool for clinical and epidemiologic research and quality assurance regarding neonatal respiratory disease.

## Background

Neonatal clinical databases have been established in most developed countries. They may contain information from a single hospital, a region, a country, or several countries. The Danish neonatal database, the NeoBase, was established in 1996, and has been used in various neonatal intensive care units (NICUs) throughout the country for variable time periods. The NeoBase was originally designed with the purpose of benchmarking nationally and internationally. For this reason the variables necessary to create critical risk score for babies (CRIBI and later CRIBII) [1,2] were collected along with variables that would facilitate comparison with data from e.g. the Vermont Oxford Network.

Respiratory problems are the most common cause of admission to NICUs, affecting 2–3% of all newborns. Many factors, including prematurity, gender, mode of delivery, and genetic predisposition, are involved in the aetiology of these problems [3]. Several studies have been based on information from the NeoBase [4,5]. Secondary data, e.g. the NeoBase, are data that have not been collected with one specific research purpose [6].

Secondary data may constitute a valuable and cost-efficient alternative to the use of primary data in epidemiologic research [7-9]. However, using secondary data has disadvantages related to the researcher’s difficulty in controlling the selection of the variables registered, participants included, and information quality [8].

We sought to evaluate the quality of the following respiratory variables in the NeoBase: surfactant administration, nasal continuous positive airway pressure (nCPAP), oxygen supplementation, and mechanical ventilation. We evaluated data from the North and Central Denmark Regions, an area with a population of 1.8 million people and about 23,000 deliveries per year.

To our knowledge, no studies have previously evaluated the data quality of respiratory variables in neonatal databases.

## Methods

The evaluation of the NeoBase included all very preterm neonates born in 2005 with a gestational age less than 32 weeks and admitted to one of the two level III NICUs in the regions: Aalborg University Hospital and Aarhus University Hospital, Skejby, Denmark.

The NeoBase contains clinical and demographic information on all newborns admitted to the NICUs in the North and Central Denmark Region. Clinicians at the neonatal departments routinely register the information on a structured form that includes birth weight, gestational age, Apgar scores, cerebral complications, and treatment of respiratory distress, pneumothorax, persistent ductus arteriosus, necrotising enterocolitis, and infections. The following data on respiratory treatment and support are registered: surfactant administration (yes/no) and duration of days of nCPAP, oxygen supplement, and mechanical ventilation. These data are collected from medical records and nurses’ charts at the time of a patient’s discharge from the hospital. At discharge all patients hospitalised in Denmark receive ICD10 codes covering the diseases and procedures during their stay in the hospital. The physician who discharges the patient typically provides these codes. All ICD10 codes are recorded electronically and stored in the Danish National Patient Register (DNPR) [10]. The ICD 10 codes are gathered from the files ultimately sent to the DNPR.

Very preterm and very sick infants admitted to NICUs are under close observation by the bedside nurse, and the observations are registered on structured charts for each infant. Because of their frequent, prospective bedside observations of the infants, the nurses’ charts were used as reference standard in the evaluation of the NeoBase. Treatment with nCPAP, oxygen, and mechanical ventilation is routinely registered hourly along with the patient’s vital signs during every shift. Surfactant administration and changes in nCPAP, oxygen, and mechanical ventilation are immediately registered. The information from the nurses’ charts was extracted by SA without knowledge of the value of the variables in the NeoBase for the particular patient.

We estimated the data quality of the dichotomous variables (treatment versus no treatment), surfactant administration, nCPAP, oxygen supplementation, and mechanical ventilation, by calculating the sensitivity, specificity, and the positive and negative predictive values of the information registered in the NeoBase [8] using the following definitions. Sensitivity: number of infants treated according to both the NeoBase and the reference standard, divided by number of infants treated according to the reference standard. Specificity: number of infants not treated according to both the NeoBase and the reference standard, divided by the number of infants not treated according to the reference standard. Positive predictive value: number of infants treated according to both the NeoBase and the reference standard, divided by the number of infants treated according to the NeoBase. Negative predictive value: number of infants not treated according to both the NeoBase and the reference standard, divided by the number of infants not treated according to the NeoBase.

Measures of association are presented with 95% confidence intervals calculated by use of the binomial function in the STATA statistical software version 12.

The continuous variables, duration of oxygen supplementation, nCPAP, and mechanical ventilation, were analysed and illustrated in accordance with Kristensen et al. [11] by studying the difference between the NeoBase and the reference standard.

Percentage of concordance was used as a measure of agreement between the NeoBase and the reference standard. For the dichotomous variables the concordance was defined as the sensitivity of the information registered in the NeoBase. For the continuous variables, the concordance was based on the discrepancy in days between the NeoBase and the reference standard. Percentage of concordance was calculated, allowing a discrepancy of 0, 1, and 2 days between the registrations. The percentage of concordance was described as high (> 90), moderate (70–90), or low (< 70) [11].

In Denmark, approval from the Ethics Commitee and the Protection agency is not mandated for local quality assurance databases and registries based on medical records and bed side registrations.

## Results

A total of 164 very preterm neonates were admitted to the two hospitals in 2005. For 11 neonates, the nurses’ charts were missing. Thus, we evaluated information from 153 (93%) patients. Demographic and clinical characteristics of the patients are shown in Table 1. Their median gestational age at birth was 28 weeks, and the median birth weight was 1128 g. According to the ICD10 code P22.0, 107 (70%) of the neonates had respiratory distress syndrome. They were treated with nCPAP, oxygen supplementation, surfactant, and if necessary mechanical ventilation. Virtually all the newborns received nCPAP from birth or following extubation. Sixty-four percent of the neonates were treated with oxygen, 33% with surfactant replacement, and 29% with mechanical ventilation.

**Table 1:** Demographic and clinical characteristics, obtained from the nurses’ charts, of 153 very preterm newborns, Aalborg and Aarhus, Denmark 2005

Gestational age (weeks), median (range)	28	(24-31)
Birth weight (g), median (range)	1128	(520-2750)
Gender		
Girls, n (%)	81	(53)
Boys, n (%)	72	(47)
Respiratory distress syndrome, n (%)*	107	(70)
Surfactant therapy, n (%)	51	(33)
Oxygen supplementation		
n (%)	98	(64)
Length (days), median (range)	3	(0-80)
nCPAP therapy		
n (%)	145	(95)
Length (days), median (range)	13	(0-72)
Mechanical ventilation		
n (%)	44	(29)
Length (days), median (range)	0	(0-35)

*obtained from the Danish National Patient Registry (ICD-10 code P22.0).

Among the 153 patients who participated in the study, information in 11 was incomplete with regard to a single variable in the reference standard. Thus, 151, 145, 152, and 153 patients were evaluated with respect to treatment with nCPAP, oxygen, surfactant, and mechanical ventilation.

**Figure 1:**
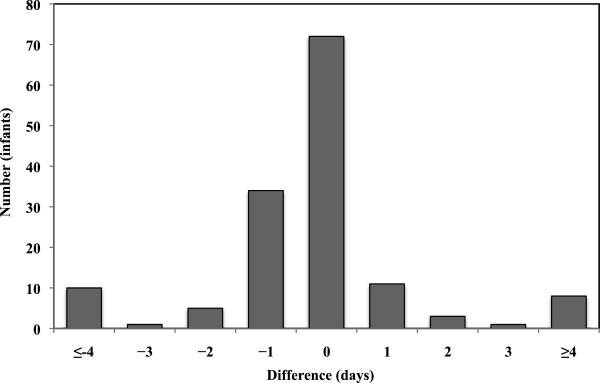
Difference in duration of nCPAP therapy between the neonatal clinical database, NeoBase and the nurses’ charts, reference standard for 145 very preterm newborns.

**Figure 2:**
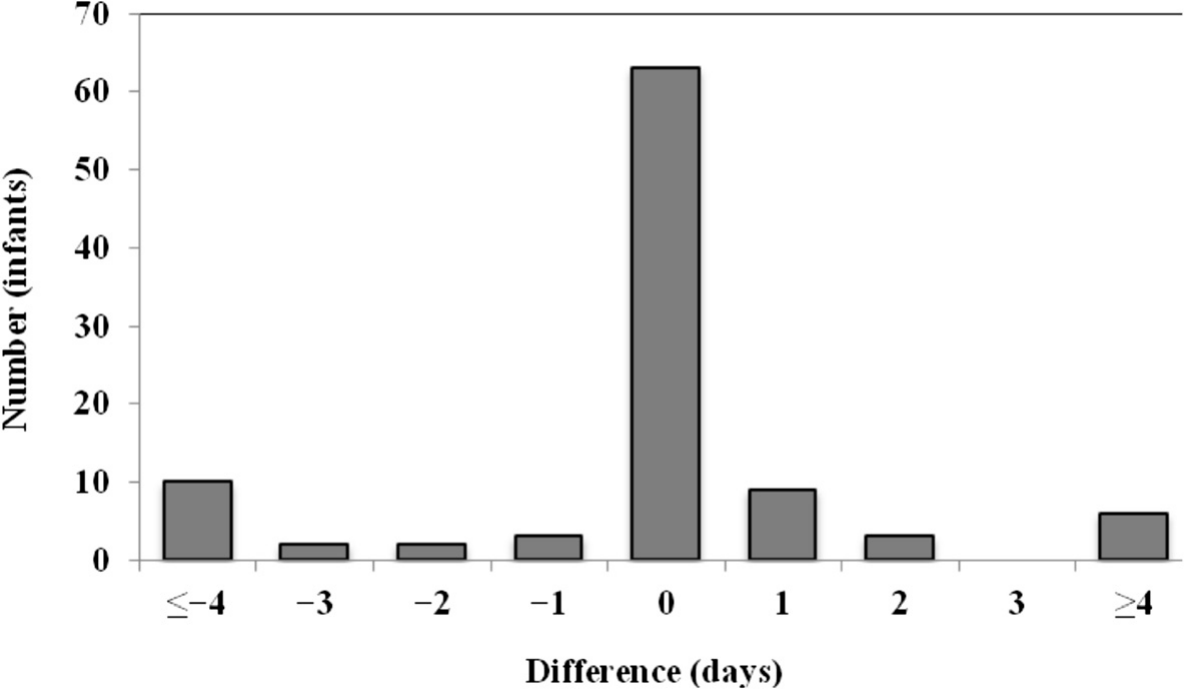
Difference in duration of oxygen supplementation between the neonatal clinical database, NeoBase and the nurses’ charts, reference standard for 98 very preterm newborns.

**Figure 3:**
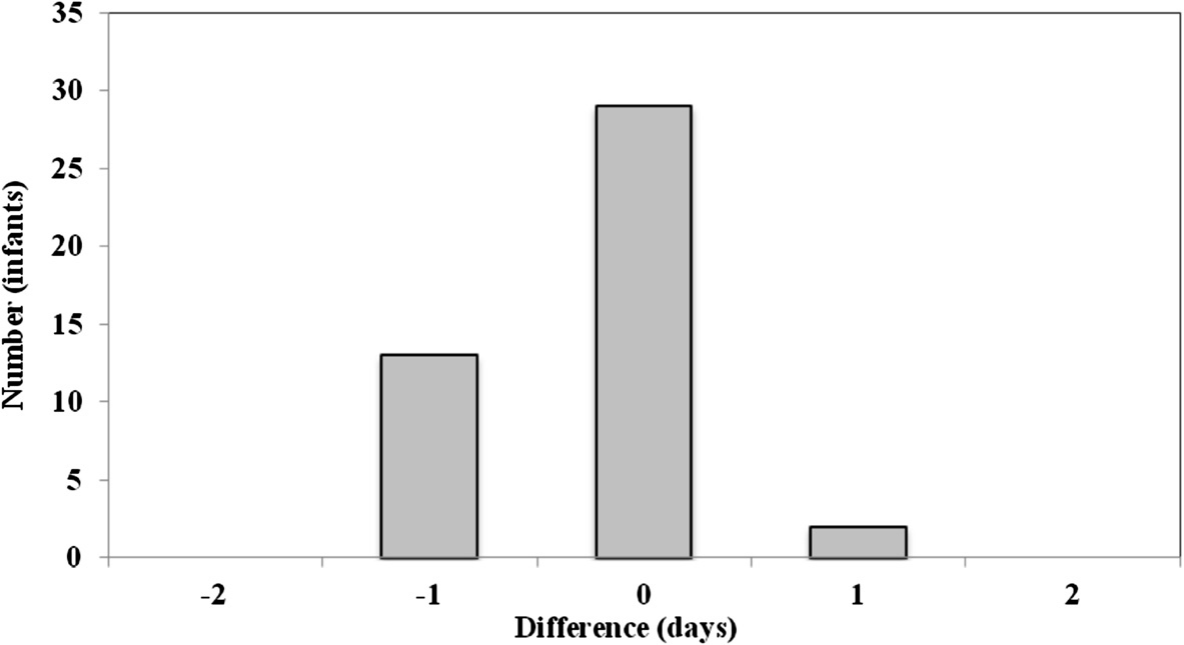
Difference in duration of mechanical ventilation between the neonatal clinical database, NeoBase and the nurses’ charts, reference standard for 44 very preterm newborns.

Evaluation of the dichotomous variables is shown in Table 2. Concordance was high for all four dichotomous variables (95–100%). The positive predictive values were 87% or higher. The evaluation of the continuous variables is seen in Figures 1, 2, 3 and Table 3. Comparing the information from the NeoBase and the reference standard, there was an underestimation of the duration of nCPAP and ventilatory therapy in the NeoBase. This phenomenon was not seen for duration of oxygen therapy. The concordance was low for all three variables if we assumed identical registration. However, we found moderate agreement for nCPAP (81%) and oxygen therapy (77%) if we allowed a discrepancy of 1 day between the NeoBase and the reference standard. Complete agreement was seen for duration of mechanical ventilation under the same conditions. Concordance was still moderate if we allowed a discrepancy of 2 days, although we found a minor improvement in concordance for nCPAP (87%) and oxygen therapy (82%). Concordance improved when we allowed the discrepancy to increase and was close to 100% when a discrepancy of 7 days in treatment was allowed.

**Table 2:** Sensitivity, specificitity, positive and negative predictive values (PPV, NPV) for the dichotomous variables related to respiratory support on comparison of the clinical database with nurses’s charts for 153 very preterm newborns, Aalborg and Aarhus, Denmark, 2005

	**Sensitivity**	**Specificity**	**PPV**	**NPV**
**Variable**	**(95% CI)**			
Surfactant therapy	100	98 (95;100)	96 (91;100)	100
nCPAP therapy	99 (98;100)	100	100	86 (60;100)
Oxygen supplementation	90 (84;96)	72 (60;85)	87 (81;94)	77 (65;90)
Mechanical ventilation	95 (89;100)	95 (91;99)	89 (81;98)	98 (96;100)

**Table 3:** Agreement between the neonatal clinical database, NeoBase and the reference standard with regard to length of nCPAP therapy, oxygen supplementation, and mechanical ventilation, by a difference of 0, 1 and 2 days

	**Concordance (%)**		
	**Difference**	**Difference**	**Difference**
Variabel	0 days	±1 day	±2 days
nCPAP therapy (n = 145)	50	81	87
Oxygen suppl. (n = 98)	62	77	82
Mechanical vent. (n = 44)	68	100	100

Following stratification by dichotomous gestational age at 28 completed weeks or less and above 28 weeks, we found no differences in the concordance of dichotomous or continuous variables. Furthermore, no differences in concordance were observed between the two hospitals (data not shown).

## Discussion

We found that concordance was high between the information from a clinical database, the NeoBase, and the reference standard (detailed bedside registration) for all four dichotomous treatment variables: surfactant administration, nCPAP, oxygen supplementation, and mechanical ventilation in very preterm newborns. Concordance was low for the continuous variables if no discrepancy was allowed. If agreement was defined as within 1 day, the concordance improved to a moderate level for duration of oxygen supplementation and nCPAP therapy. Complete agreement was seen for duration of mechanical ventilation under the same conditions.

An advantage of the study was the use of the nurses’ charts as reference standard in the evaluation of the NeoBase. Nurses chart all vital signs, such as oxygen saturation, respiratory frequency, blood pressure, heart rate, and any changes in these, along with treatment on a structured chart throughout their shifts in accordance with the chart’s time schedule or doctors’ orders. The observations are current and frequent. Thus, the evaluation of the NeoBase was based on a reference standard validated by use in the clinical setting and during the care of the newborn. This information is also used by the medical staff on rounds and whenever the infant’s condition deteriorates. Thus, errors are likely to be few and unrelated to hospital or specific newborns. Furthermore, the information from the nurses’ charts for this particular study was extracted by the same person (SA) without knowledge of the value of the variables in the NeoBase for the particular patient. Thus, there was no inter-individual variation in the extraction of data from the charts.

A limitation of the study was that the evaluation was based on a relatively small study population. However, the confidence intervals for the sensitivities and the positive predictive values calculated were quite small for the dichotomous variables.

The concordance may vary according to specific characteristics related to the infant or the way chart or database information is gathered, i.e. neonates of extremely low gestational age may be more closely monitored or monitored in a different way than neonates of higher gestational age. Also, the concordance may have been influenced by NICU-specific registrations. However, we found that the concordance was unrelated to gestational age and hospital.

The quality of the data in the clinical databases may depend on the workload of the clinician who completed the form for the database as well as the clinician’s motivation. The motivation may depend on the degree of feedback of information from the database. In both NICUs, completion of the forms was mandatory at the time of discharge of the newborn. However, feedback to the clinicians was only carried out sporadically. We were unable to identify periods with heavy workload to rule out whether this may have influenced validity.

Considering treatment and duration of oxygen and nCPAP therapy, the quality of registered data may depend on the level of the NICU, being lower from level III than from level II units due to more complicated patients. Both units included in this study were level III units. Therefore we expect validity for other units in Denmark to be as good or even better for some of the variables tested.

## Conclusions

We conclude that the registration of variables related to newborn respiratory morbidity in the clinical database of newborns in Denmark, the NeoBase, had a quality that makes the NeoBase a valuable tool for clinical and epidemiologic research and quality assurance. Thus, simple and structured registration by physicians with a high workload can be used for these purposes.

## Abbreviations

nCPAP: Nasal continuous positive airway pressure; NeoBase: Danish neonatal clinical database; NICU: Neonatal intensive care unit; PPV: Positive predictive value; NPV: Negative predictive value.

## Competing interests

The publication is funded by The Danish Council for Independent Research. The funder did not have any role in the study design, analysis, interpretation or dissemination of research findings. The authors declare that they have no competing interests.

## Authors’ contributions

JPP, TBH, and FE contributed to the conception and design of the study. SA extracted data and conducted the analysis, with support from JPP, TBH, and FE. The first draft of the manuscript was written by SA and FE. All authors contributed to data interpretation and critical revision of the manuscript. All authors have read and approved the final version submitted.

## Pre-publication history

The pre-publication history for this paper can be accessed here:

http://www.biomedcentral.com/1471-2431/14/47/prepub
